# Authors’ response on Hoppe et al. (2015) “Effects of a neonicotinoid pesticide on honey bee colonies: a response to the field study by Pilling et al. (2013)”. Environ Sci Eur (2015) 27–28

**DOI:** 10.1186/s12302-015-0064-3

**Published:** 2015-11-23

**Authors:** Peter Campbell, Mike Coulson, Natalie Ruddle, Ingo Tornier, Ed Pilling

**Affiliations:** 1 Jealotts Hill International Research Centre, Syngenta, Bracknell, Berkshire RG42 6EY UK; 2 Eurofins, Eutinger Str. 24, 75223 Niefern-Öschelbronn, Germany; 3 JSC International Limited, The Exchange, Station Parade, Harrogate, North Yorkshire HG1 1TS UK

**Keywords:** Thiamethoxam, Honeybee, Field studies, Critical review

## Abstract

The published Commentary by Hoppe et al. (Environ Sci Eur 27–28, 2015) makes a number of strong criticisms of Pilling et al. (PLoS One 8:e77193, 2013), which this authors’ response will show are either wrong, inaccurate or misleading. A selection of these misrepresentations include a claim that technical thiamethoxam was used rather than the commercial product. This is not true. Pilling et al. (PLoS One 8:e77193, 2013) clearly state that formulated commercial products were used which also included fungicides. It is claimed that there was a failure to quantify colony losses in winter. Again this is not true. These data were readily extractable from the original paper. It is claimed that 70 % of the colonies did not survive. For a multiple year study this is very misleading. The annual colony loss rate was 14.8 % for treated colonies and 16 % for control colonies, well within background colony loss rates reported by the EC Epilobee EU monitoring programme. Concerns are also raised regarding the *PLOS One* reviewing process. The reality is that Pilling et al. (PLoS One 8:e77193, 2013) was extensively reviewed by five referees during the original review process, followed by a second post-publication editorial review. These inaccurate and misleading statements are then used to infer that the data, methodology and conclusions of low risk to honeybees from Pilling et al. (PLoS One 8:e77193, 2013) are untruthful and misleading. This inference is both absolutely untrue and inappropriate. Pilling et al.’s (PLoS One 8:e77193, 2013) paper is a summary of one the most comprehensive set of field studies ever conducted on honeybees, a fact recognised within both the second review by *PLOS One* and within the published EFSA Evaluation of Thiamethoxam.

## Background

In response to the increasing call for industry to be more transparent, Syngenta took the decision to publish the pivotal honeybee field studies that supported the honey bee safety of thiamethoxam. These field studies included 12 separate pollen and nectar field residue trials and five long-term (4 consecutive years) field effects studies on honeybees carried out in four geographically widespread locations in France. The primary purpose of these field trials was to investigate and test the bee safety of thiamethoxam. As such these studies have to be carefully designed to avoid as far as is possible any other potential confounding factors, e.g. exposure to other insecticides. In response to the criticism of Hoppe et al. (2105) that the “design and adherence to the protocol was described inadequately” and that it was “doubtful whether the study was implemented in a traceable way”, it should be noted that these studies also have to comply with the strict, legally enforceable, quality control requirements of Good Laboratory Practice (OECD [1]) and comply with relevant international guidelines, e.g. in this case EPPO 170, 2010 [2]. One of the challenges faced during the drafting of the publication of these field trials was how to summarise in a concise publication manuscript the extensive methodology information from effectively 13 separate field trials as well as the vast amount of data that were generated. Initially two separate papers were drafted to summarise the data on residues in pollen and nectar and the other on effects on honeybee colonies. However, *PLOS One* insisted that they be combined into a single manuscript. In the end, the final Pilling et al.’s [3] paper was 13 pages long with a substantial supplementary information section. Whilst many of the criticisms made by Hoppe et al. [4] can be addressed by a more thorough reading of the original Pilling et al.’s [3] paper, the other criticisms, particularly of the methodology used, are fully addressed in the original study reports for these field studies which were submitted to EFSA and other Regulatory Authorities. One of the comments made by this EFSA evaluation (EFSA 2013, [5]) of these field studies was “The study was well performed and was to GLP. The study was very comprehensive and was scientifically sound.” (See comment on Hecht-Rost 2009 study on page 39 of Doc 3067prr in EFSA link http://registerofquestions.efsa.europa.eu/roqFrontend/outputLoader?output=ON-3067). A detailed authors’ response to each of the points raised by Hoppe et al. [4] is listed below:

## Detailed comments on Hoppe et al. [4]

### EC neonicotinoid restriction in use

Hoppe et al. [4] quotes “a 2 yr Moratorium implemented by the European Commission”. This again is incorrect. The restriction on neonicotinoids implemented by European Commission is not time bound to 2 years.

### Usage of non-commercial product for experiments

In [4], Hoppe et al. claim that “the active ingredient only was used for seed dressing and not a commercial product”. This statement is wrong. Pilling et al.’s [3] paper clearly states that formulated products “as per normal seed treatment practice” were used, which did for example include the use of the fungicides, metalaxyl-M and fludioxonil. Therefore, all the text that follows under this point is not relevant and based on a wrong assumption.

### Hoppe et al. [4] point out that Pilling et al.’s [3] study is carried out at a lower application rate than the maximum recommended application rate

Pilling et al.’s [3] study was designed to only address the pre-dominant commercial rates that were most commonly used in Europe for these crops. The higher hectare rates registered in few EU countries at the time of the EFSA review, which exceeded the rates tested in the studies described in Pilling et al. (2013), were predominantly the result of outdated old seeding rates and not in line with current realistic seeding rates in Europe. Another important point that is missed by Hoppe et al. [4] is that the most important and relevant application rate for a risk assessment, investigating the effect of systemic residues of thiamethoxam in pollen and nectar from a seed treatment application, is actually the seed loading rate (i.e. g active ingredient/seed) not the amount of active ingredient applied per hectare, i.e. it is the seed loading that will drive the systemic residue concentration of the active ingredient in the pollen and nectar not the application rate per hectare.

### Separation distance of 2 km between treatment and control

Whilst Pilling et al. [3] do quote “about 2 km” as the separation difference, this was the minimum target and in the reality of the trials, this was often exceeded.

### No evidence to support the isolation of trials from other bee attractive crops

Whilst no detailed location information is presented in Pilling et al. [3], the latitude and longitude coordinates are available for all the test and control sites within the full study reports submitted to EFSA. Field surveys were also carried out at farm level each year prior to trial commencing to confirm that the sites were isolated from bee attractive crops for the duration of the exposure phase.

### Time window of exposure

Hoppe et al. [4] criticise the time window of exposure being too short and “not field relevant”. The fact is that this study was specifically investigating the risk from systemic residues in pollen and nectar from thiamethoxam-treated crops in flower. So the time window of exposure in Pilling et al. [3] was for the complete flowering period of the crop for each of the plots being monitored. The duration of the flowering was dependent on local environmental conditions. One would question how a complete flowering period of a commercially planted crop cannot be field relevant to investigating risk from systemic residues in pollen and nectar?

### Use of woodland sites

Hoppe et al. [4] state that keeping the colonies in woodland sites “without intensive agricultural crops” does not reflect normal beekeeping practice. However, for the purpose of investigating whether systemic residues in pollen and nectar from flowering crops seed treated with thiamethoxam present any risk to honeybee colonies, it is absolutely necessary to isolate the experiment from any other confounding pesticide inputs during the post flowering period. For this reason, the post flowering sites had to be located away from other agricultural fields. In fact, if the colonies had been located close to other bee attractive agricultural crops (as may occur in beekeeping) the results could be challenged based on dilution of residues. Latitude and longitude coordinates of the over-wintering sites were not included in Pilling et al. [3] for reasons of brevity but are available in raw data. Field surveys were also carried out prior to selection of over-wintering sites.

### Pesticide contamination history of sites

Hoppe et al. [4] state that pesticide contamination history of site was not assessed. Whilst the pesticide contamination history information for each site was not stated in Pilling et al. [3] (for brevity), it was assessed for 3 years prior to the start of the experiment and was included in the full study reports submitted to EFSA.

### Residue analysis

Residue analysis was only carried out for thiamethoxam and its metabolite since these were the test items being studied and every effort was made to prevent exposure of colonies to any other pesticides during the exposure period, e.g. through implementation of isolation distances and over-wintering sites. Other pesticides were applied as necessary outside the flowering period (and hence outside the exposure period of trial) to ensure a viable crop.

### Cumulative colony mortality

This quoted figure of 70 % total colonies lost in Hoppe et al. [4] is completely misleading. This was a 4-year trial so the most appropriate way of looking at the colony loss data is to express the number of colonies lost per year. Since this is how EC Honeybee Colony Loss Monitoring surveys, such as EPILOBEE (Laurent et al. [6]), express the national colony loss statistics for Europe. The data from Pilling et al. [3] indicate an annual colony loss of 14.8 % per year for treated colonies and 16 % for control colonies. This is well within the normal range experienced by European Beekeepers and the numbers of colonies lost were similar across treatment and controls. The “male brood only” effects that are discussed and attributed to thiamethoxam in Hoppe et al. [4] occurred equally in control and treatment colonies.

### Colony loss data in the winter

Hoppe et al. [4] state that “no data were reported for colony losses during the winter”. They also go on to present different colony loss numbers from Pilling et al. [3] based on their analysis of “raw data” although we are unclear where these data originated. Although Pilling et al. [3] did not separate out over-wintering colony loss data, it is easily calculated from Table 2 within the original paper. However, for clarity we have separated out over-wintering and total colony loss data below in Table 1.

**Table 1:** Total colony losses and over-wintering colony losses represented from Table 2 in Pilling et al. (2013) [includes colonies lost to AFB (1 from the control group and 3 from the treated group at a single site in 2007)] [2]

	Control		Treated	
	Total	Over-winter	Total	Over-winter
Maize, *n* = 18				
2006	0	0	1	0
2007	1	0	8	2
2008	3	0	7	1
2009	7	1	1	1
2010	6	6	4	4
Total	17	7	21	8
Average per year	4.25	1.4	4.2	1.6
Average % colonies	23.6	7.8	23.3	8.9
Oilseed rape, *n* = 12				
2005	1	0	0	0
2006	0	0	0	0
2007	0	0	3	3
2008	2	2	0	0
2009	1	1	0	0
Total	4	3	3	3
Average per year	1	0.75	0.75	0.75
Average % colonies	8.33	6.25	6.25	6.25
Overall %	16.0	7.0	14.8	7.6

As can be seen from Table 1, the control and treatment colonies showed a similar pattern of both total and over-wintering colony losses. Also, the male brood only effects referenced by Hoppe et al. (2015) are equally present in both control and treated colonies. So once again the inference that we are hiding or misrepresenting data that show a treatment-related effect of thiamethoxam is fundamentally incorrect.

### Rundlof et al.’s [7] study

Hoppe et al. [4] compare the methodology of Pilling et al. (2013) with the more recent Rundlof et al.’s [7] study, suggesting the former study has more deficiencies. However, what they fail to point out is that the honeybee results for these two sets of field studies were the same, i.e. there were no detectable effects of the neonicotinoid seed treatment on honeybee colonies.

### Lack of statistical analysis

Hoppe et al. [4] criticise Pilling et al. [3] for a lack of statistical analysis. The difficulty of including sufficient statistical replication in the design of large-scale honey bee field trials, such as this, is well described in the both the original Pilling et al.’s [3] paper and in the EPPO [2] Field testing Guideline followed. Both clearly state that although replication is desirable it is not practically feasible because of the isolation requirements of the study design. This aspect is discussed very clearly in Pilling et al. [3] and was accepted by the Journal.

### Inadequate refereeing process

Hoppe et al. [4] question *PLOS One’s* decision to publish Pilling et al.’s [3] paper, based on the above list of criticisms (most of which we have now shown are either incorrect or unjustified). However, it should be noted that Pilling et al.’s [3] paper was thoroughly peer reviewed by an academic editor and five different reviewers prior to publication. Furthermore, following comments received by the Journal after publication, *PLOS One* carried out a second additional review of this paper, by a member of the *PLOS One* editorial board. Once again this paper was accepted as stands (see link to detailed comments from this second review http://www.plosone.org/annotation/listThread.action?root=82356). One of the comments made by the editor during this second review was “The effort was comprehensive and seems honestly described.”

## Conclusions

We contend that the alleged deficiencies claimed by Hoppe et al. [4] to undermine the conclusions of Pilling et al. [3] are incorrect, unjustified or are clear misunderstandings of the design, conduct and purpose of the original regulatory field studies, which could have been easily addressed by clarification with the authors.
